# Bis(4-cyano-1-methyl­pyridinium) bis­(1,2-dicyano­ethene-1,2-dithiol­ato-κ^2^ *S*,*S*′)cuprate(II)

**DOI:** 10.1107/S1600536812001377

**Published:** 2012-01-18

**Authors:** Na Wang, Jin-Guo Wang, An-Jie Min, Yao-Wen Fu

**Affiliations:** a First Hospital Affiliated to Jilin University, Changchun 130021, People’s Republic of China; b Xiangya Hospital, Central South University, Changsha 410008, People’s Republic of China

## Abstract

The title ion-pair compound, (C_7_H_7_N_2_)_2_[Cu(C_4_N_2_S_2_)_2_], was obtained by the direct reaction of CuCl_2_·2H_2_O, disodium maleonitrile­dithiol­ate (Na_2_mnt) and 4-cyano-1-methyl­pyridinium iodide. The anion and one pyridinium cation lie entirely on a mirror plane, whereas for the other cation, a crystallographic mirror plane runs through the N and *para*-C atoms of the pyridine ring, the methyl C atom, and the cyano group. In the crystal, ions are linked into a three-dimensional network by C—H⋯N hydrogen bonds.

## Related literature

For details of other square-planar *M*(dithiol­ene)_2_ complexes, see: Robin & Fromm (2006 ▶); Nishijo *et al.* (2003 ▶); Robertson & Cronin (2002 ▶); Coomber *et al.* (1996 ▶); Duan *et al.* (2010 ▶). For a study on CN⋯π inter­actions, see: Tian *et al.* (2007 ▶).
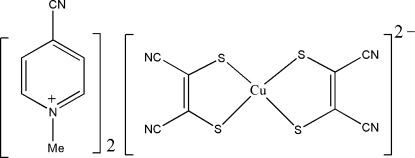


## Experimental

### 

#### Crystal data

(C_7_H_7_N_2_)_2_[Cu(C_4_N_2_S_2_)_2_]
*M* *_r_* = 582.19
Monoclinic, 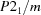

*a* = 12.063 (2) Å
*b* = 6.9282 (14) Å
*c* = 15.118 (3) Å
β = 91.530 (3)°
*V* = 1263.0 (4) Å^3^
*Z* = 2
Mo *K*α radiation
μ = 1.22 mm^−1^
*T* = 291 K
0.20 × 0.18 × 0.12 mm

#### Data collection

Bruker SMART APEX CCD area-detector diffractometer
Absorption correction: multi-scan (*SADABS*; Bruker, 2000 ▶) *T*_min_ = 0.784, *T*_max_ = 0.863
6296 measured reflections
2418 independent reflections
1717 reflections with *I* > 2σ(*I*)
*R*_int_ = 0.090

#### Refinement

*R*[*F* ^2^ > 2σ(*F* ^2^)] = 0.041
*wR*(*F* ^2^) = 0.101
*S* = 1.00
2418 reflections
205 parameters
H-atom parameters constrained
Δρ_max_ = 0.32 e Å^−3^
Δρ_min_ = −0.50 e Å^−3^

### 

Data collection: *SMART* (Bruker, 2000 ▶); cell refinement: *SAINT* (Bruker, 2000 ▶); data reduction: *SAINT*; program(s) used to solve structure: *SHELXS97* (Sheldrick, 2008 ▶); program(s) used to refine structure: *SHELXL97* (Sheldrick, 2008 ▶); molecular graphics: *SHELXTL* (Sheldrick, 2008 ▶); software used to prepare material for publication: *SHELXTL*.

## Supplementary Material

Crystal structure: contains datablock(s) I, global. DOI: 10.1107/S1600536812001377/rz2689sup1.cif

Structure factors: contains datablock(s) I. DOI: 10.1107/S1600536812001377/rz2689Isup2.hkl

Additional supplementary materials:  crystallographic information; 3D view; checkCIF report

## Figures and Tables

**Table 1:** Hydrogen-bond geometry (Å, °)

*D*—H⋯*A*	*D*—H	H⋯*A*	*D*⋯*A*	*D*—H⋯*A*
C1—H1*A*⋯N8^i^	0.93	2.60	3.533 (6)	179
C2—H2*A*⋯N7^i^	0.93	2.44	3.309 (6)	156
C5—H5*A*⋯N4^ii^	0.93	2.36	3.247 (6)	159
C8—H8*A*⋯N2^iii^	0.93	2.48	3.196 (5)	134
C9—H9*A*⋯N5^iv^	0.93	2.51	3.297 (4)	143

Symmetry codes: (i) 
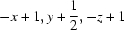
; (ii) 
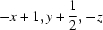
; (iii) 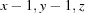; (iv) 
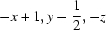
.

**Fig. 1.**
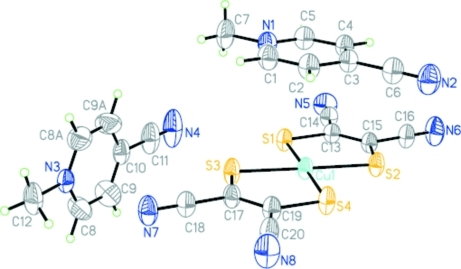
The molecular structure of the title compound, showing 30% probability displacement ellipsoids [symmetry code: (A) x, 0.5-y, z].

## supplementary crystallographic information

### Comment

During the past few years, 1,2-dithiolene metal complexes have been important molecular materials with interesting physical properties, such as electrical conductivity, superconductivity, magnetic and non-linear optic properties (Robertson & Cronin, 2002; Coomber *et al.*, 1996; Robin & Fromm, 2006; Nishijo *et al.*, 2003; Duan *et al.*, 2010). Maleonitriledithiolate (mnt^2-^) transition metal complexes are a series of bis-1,2-dithiolene complexes showing such properties. Herein, we report the synthesis and crystal structure of a new Cu(mnt)_2_^2-^ salt containing the 4-cyano-1-methylpyridinium (MeCyPy)^+^ cation.

The asymmetric unit of the title compound (Fig. 1) contains (MeCyPy)^+^ cations and Cu(mnt)_2_^2-^ anions in the molar ratio 2:1. The anion and one cation (N1/N2(C1–C7) lie entirely on a mirror plane, whereas the other cation (N3/N4/C8–C12) has crystallographically imposed mirror symmetry, the mirror plane running through the N and *para*-C atoms of the pyridine ring, the methyl C atom, and the cyano group. In the crystal structure, relatively short CN···π contacts along the *a* axis [N7···Cg1 = 3.399 (3) Å; Cg1 is the centroid of the pyridine ring containing atoms N3, C8–C10] (Tian *et al.*, 2007) and longer S···π contacts along *b* axis [S1···Cg2^i^ = 3.789 (7) Å; Cg2 is the centroid of the N1/C1–C5 ring; symmetry code: (i) x, -1+y, z] are observed. The crystal packing is stabilized by C—H···N hydrogen bonds (Table 1) linking cations and anions into a three-dimensional network.

### Experimental

The title compound was prepared by the direct reaction of CuCl_2_.2H_2_O (1 mmol), disodium maleonitriledithiolate (2 mmol) and 4-cyano-1-methylpyridinium iodide (2 mmol) in an ethanol/H_2_O (1:1 *v*/*v*) solution. After filtration, the crude product was dissolved in CH_3_CN. Red-brown block-like single crystals were obtained after about two weeks on slow evaporation of the solvents at room temperature.

### Refinement

All H atoms were fixed geometrically and treated as riding with C—H = 0.93–0.96 Å, and with *U*_iso_(H) = 1.2*U*_eq_(C) or 1.5*U*_eq_(C) for methyl H atoms. The H atoms bound to the C12 methyl carbon atom are disordered over two sites about a mirror plane with site occupancies of 0.5.

### Crystal data

**Table:**

(C_7_H_7_N_2_)_2_[Cu(C_4_N_2_S_2_)_2_]	*F*(000) = 590
*M**_r_* = 582.19	*D*_x_ = 1.531 Mg m^−^^3^
Monoclinic, *P*2_1_/*m*	Mo *K*α radiation, λ = 0.71073 Å
Hall symbol: -P 2yb	Cell parameters from 2205 reflections
*a* = 12.063 (2) Å	θ = 2.7–26.5°
*b* = 6.9282 (14) Å	µ = 1.22 mm^−^^1^
*c* = 15.118 (3) Å	*T* = 291 K
β = 91.530 (3)°	Block, brown-red
*V* = 1263.0 (4) Å^3^	0.20 × 0.18 × 0.12 mm
*Z* = 2	

### Data collection

**Table:**

Bruker SMART APEX CCD area-detector diffractometer	2418 independent reflections
Radiation source: sealed tube	1717 reflections with *I* > 2σ(*I*)
graphite	*R*_int_ = 0.090
φ and ω scans	θ_max_ = 25.0°, θ_min_ = 2.1°
Absorption correction: multi-scan (*SADABS*; Bruker, 2000)	*h* = −14→13
*T*_min_ = 0.784, *T*_max_ = 0.863	*k* = −8→7
6296 measured reflections	*l* = −17→16

### Refinement

**Table:**

Refinement on *F*^2^	Primary atom site location: structure-invariant direct methods
Least-squares matrix: full	Secondary atom site location: difference Fourier map
*R*[*F*^2^ > 2σ(*F*^2^)] = 0.041	Hydrogen site location: inferred from neighbouring sites
*wR*(*F*^2^) = 0.101	H-atom parameters constrained
*S* = 1.00	*w* = 1/[σ^2^(*F*_o_^2^) + (0.0386*P*)^2^] where *P* = (*F*_o_^2^ + 2*F*_c_^2^)/3
2418 reflections	(Δ/σ)_max_ < 0.001
205 parameters	Δρ_max_ = 0.32 e Å^−^^3^
0 restraints	Δρ_min_ = −0.50 e Å^−^^3^

### Special details

**Table:**

Geometry. All e.s.d.'s (except the e.s.d. in the dihedral angle between two l.s. planes) are estimated using the full covariance matrix. The cell e.s.d.'s are taken into account individually in the estimation of e.s.d.'s in distances, angles and torsion angles; correlations between e.s.d.'s in cell parameters are only used when they are defined by crystal symmetry. An approximate (isotropic) treatment of cell e.s.d.'s is used for estimating e.s.d.'s involving l.s. planes.
Refinement. Refinement of *F*^2^ against ALL reflections. The weighted *R*-factor *wR* and goodness of fit *S* are based on *F*^2^, conventional *R*-factors *R* are based on *F*, with *F* set to zero for negative *F*^2^. The threshold expression of *F*^2^ > σ(*F*^2^) is used only for calculating *R*-factors(gt) *etc*. and is not relevant to the choice of reflections for refinement. *R*-factors based on *F*^2^ are statistically about twice as large as those based on *F*, and *R*- factors based on ALL data will be even larger.

### Fractional atomic coordinates and isotropic or equivalent isotropic displacement parameters (Å^2^)

**Table:**

	*x*	*y*	*z*	*U*_iso_*/*U*_eq_	Occ. (<1)
C1	0.6179 (4)	0.7500	0.2599 (3)	0.0729 (13)	
H1A	0.5519	0.7500	0.2903	0.087*	
C2	0.7158 (4)	0.7500	0.3051 (3)	0.0713 (12)	
H2A	0.7173	0.7500	0.3667	0.086*	
C3	0.8129 (3)	0.7500	0.2605 (2)	0.0607 (11)	
C4	0.8091 (3)	0.7500	0.1692 (2)	0.0617 (11)	
H4A	0.8741	0.7500	0.1374	0.074*	
C5	0.7088 (4)	0.7500	0.1267 (2)	0.0664 (12)	
H5A	0.7051	0.7500	0.0652	0.080*	
C6	0.9162 (4)	0.7500	0.3084 (3)	0.0741 (13)	
C7	0.5079 (4)	0.7500	0.1235 (3)	0.1021 (17)	
H7A	0.4495	0.7500	0.1653	0.153*	
H7B	0.5019	0.8631	0.0870	0.153*	
C8	0.1754 (3)	0.0879 (5)	0.3350 (2)	0.0932 (11)	
H8A	0.1563	−0.0273	0.3623	0.112*	
C9	0.2320 (3)	0.0829 (5)	0.2571 (2)	0.0982 (12)	
H9A	0.2510	−0.0344	0.2317	0.118*	
C10	0.2595 (3)	0.2500	0.2183 (2)	0.0680 (12)	
C11	0.3235 (4)	0.2500	0.1392 (3)	0.1017 (19)	
C12	0.0896 (4)	0.2500	0.4554 (3)	0.0953 (17)	
H12A	0.0752	0.3806	0.4730	0.143*	0.50
H12B	0.1349	0.1877	0.5001	0.143*	0.50
H12C	0.0208	0.1817	0.4482	0.143*	0.50
C13	0.8020 (3)	0.2500	0.0685 (2)	0.0554 (10)	
C14	0.7986 (3)	0.2500	−0.0259 (3)	0.0646 (11)	
C15	0.8992 (3)	0.2500	0.1138 (2)	0.0552 (10)	
C16	1.0025 (4)	0.2500	0.0683 (2)	0.0690 (12)	
C17	0.5487 (3)	0.2500	0.4089 (2)	0.0583 (10)	
C18	0.4441 (3)	0.2500	0.4523 (2)	0.0638 (11)	
C19	0.6436 (3)	0.2500	0.4574 (2)	0.0612 (11)	
C20	0.6390 (3)	0.2500	0.5519 (3)	0.0728 (13)	
Cu1	0.72603 (3)	0.2500	0.26339 (3)	0.0546 (2)	
N1	0.6152 (3)	0.7500	0.1718 (2)	0.0664 (9)	
N2	0.9963 (4)	0.7500	0.3493 (3)	0.0960 (13)	
N3	0.1483 (2)	0.2500	0.3706 (2)	0.0610 (9)	
N4	0.3757 (4)	0.2500	0.0790 (3)	0.139 (2)	
N5	0.7914 (4)	0.2500	−0.1020 (2)	0.0927 (13)	
N6	1.0837 (3)	0.2500	0.0328 (2)	0.0997 (14)	
N7	0.3600 (3)	0.2500	0.4858 (2)	0.0807 (12)	
N8	0.6352 (3)	0.2500	0.6273 (2)	0.0951 (13)	
S1	0.67434 (8)	0.2500	0.11804 (6)	0.0613 (3)	
S2	0.90748 (8)	0.2500	0.22849 (6)	0.0623 (3)	
S3	0.54354 (8)	0.2500	0.29465 (6)	0.0699 (4)	
S4	0.77358 (8)	0.2500	0.41093 (6)	0.0713 (4)	

### Atomic displacement parameters (Å^2^)

**Table:**

	*U*^11^	*U*^22^	*U*^33^	*U*^12^	*U*^13^	*U*^23^
C1	0.060 (3)	0.102 (4)	0.057 (3)	0.000	0.017 (2)	0.000
C2	0.067 (3)	0.105 (4)	0.042 (2)	0.000	0.011 (2)	0.000
C3	0.054 (2)	0.083 (3)	0.046 (2)	0.000	0.0019 (18)	0.000
C4	0.053 (2)	0.085 (3)	0.048 (2)	0.000	0.0102 (18)	0.000
C5	0.065 (3)	0.092 (3)	0.042 (2)	0.000	0.004 (2)	0.000
C6	0.067 (3)	0.105 (4)	0.051 (3)	0.000	0.006 (2)	0.000
C7	0.059 (3)	0.144 (5)	0.102 (4)	0.000	−0.019 (3)	0.000
C8	0.098 (3)	0.080 (3)	0.102 (3)	−0.019 (2)	0.028 (2)	0.003 (2)
C9	0.118 (3)	0.089 (3)	0.089 (3)	−0.011 (2)	0.028 (2)	−0.029 (2)
C10	0.044 (2)	0.116 (4)	0.044 (2)	0.000	−0.0071 (18)	0.000
C11	0.058 (3)	0.191 (6)	0.056 (3)	0.000	−0.015 (2)	0.000
C12	0.057 (3)	0.163 (5)	0.066 (3)	0.000	0.015 (2)	0.000
C13	0.051 (2)	0.076 (3)	0.039 (2)	0.000	0.0050 (17)	0.000
C14	0.059 (3)	0.088 (3)	0.046 (2)	0.000	0.001 (2)	0.000
C15	0.045 (2)	0.081 (3)	0.040 (2)	0.000	0.0070 (17)	0.000
C16	0.048 (2)	0.116 (4)	0.043 (2)	0.000	0.0027 (19)	0.000
C17	0.041 (2)	0.091 (3)	0.043 (2)	0.000	0.0052 (17)	0.000
C18	0.049 (2)	0.104 (3)	0.038 (2)	0.000	0.0008 (18)	0.000
C19	0.047 (2)	0.098 (3)	0.039 (2)	0.000	0.0052 (17)	0.000
C20	0.044 (2)	0.122 (4)	0.052 (3)	0.000	−0.0014 (19)	0.000
Cu1	0.0387 (3)	0.0830 (4)	0.0423 (3)	0.000	0.0045 (2)	0.000
N1	0.055 (2)	0.085 (3)	0.059 (2)	0.000	0.0019 (17)	0.000
N2	0.075 (3)	0.139 (4)	0.074 (3)	0.000	−0.010 (2)	0.000
N3	0.0400 (18)	0.087 (3)	0.056 (2)	0.000	−0.0011 (15)	0.000
N4	0.064 (3)	0.299 (7)	0.053 (2)	0.000	−0.006 (2)	0.000
N5	0.112 (3)	0.119 (3)	0.047 (2)	0.000	0.004 (2)	0.000
N6	0.061 (3)	0.177 (4)	0.062 (2)	0.000	0.022 (2)	0.000
N7	0.052 (2)	0.139 (4)	0.052 (2)	0.000	0.0095 (17)	0.000
N8	0.076 (3)	0.165 (4)	0.044 (2)	0.000	0.0012 (19)	0.000
S1	0.0427 (6)	0.0950 (8)	0.0461 (5)	0.000	0.0003 (4)	0.000
S2	0.0401 (5)	0.1060 (8)	0.0409 (5)	0.000	0.0018 (4)	0.000
S3	0.0391 (5)	0.1299 (10)	0.0408 (5)	0.000	0.0024 (4)	0.000
S4	0.0391 (6)	0.1282 (10)	0.0465 (6)	0.000	0.0005 (4)	0.000

### Geometric parameters (Å, °)

**Table:**

C1—N1	1.331 (5)	C12—N3	1.481 (5)
C1—C2	1.350 (6)	C12—H12A	0.9600
C1—H1A	0.9300	C12—H12B	0.9600
C2—C3	1.367 (6)	C12—H12C	0.9600
C2—H2A	0.9300	C13—C15	1.342 (5)
C3—C4	1.380 (5)	C13—C14	1.428 (5)
C3—C6	1.424 (6)	C13—S1	1.729 (4)
C4—C5	1.354 (6)	C14—N5	1.151 (5)
C4—H4A	0.9300	C15—C16	1.440 (5)
C5—N1	1.334 (5)	C15—S2	1.734 (3)
C5—H5A	0.9300	C16—N6	1.129 (5)
C6—N2	1.134 (6)	C17—C19	1.343 (5)
C7—N1	1.470 (5)	C17—C18	1.437 (5)
C7—H7A	0.9589	C17—S3	1.727 (4)
C7—H7B	0.9600	C18—N7	1.146 (5)
C8—N3	1.292 (4)	C19—C20	1.431 (5)
C8—C9	1.378 (4)	C19—S4	1.735 (4)
C8—H8A	0.9300	C20—N8	1.142 (4)
C9—C10	1.343 (4)	Cu1—S3	2.2638 (11)
C9—H9A	0.9300	Cu1—S2	2.2652 (11)
C10—C9^i^	1.343 (4)	Cu1—S1	2.2679 (11)
C10—C11	1.441 (6)	Cu1—S4	2.2883 (11)
C11—N4	1.120 (6)	N3—C8^i^	1.292 (4)
			
N1—C1—C2	120.3 (4)	H12B—C12—H12C	109.5
N1—C1—H1A	119.9	C15—C13—C14	120.7 (3)
C2—C1—H1A	119.9	C15—C13—S1	123.7 (3)
C1—C2—C3	120.0 (4)	C14—C13—S1	115.5 (3)
C1—C2—H2A	120.0	N5—C14—C13	177.3 (5)
C3—C2—H2A	120.0	C13—C15—C16	120.8 (3)
C2—C3—C4	119.2 (4)	C13—C15—S2	122.4 (3)
C2—C3—C6	119.9 (4)	C16—C15—S2	116.8 (3)
C4—C3—C6	120.9 (3)	N6—C16—C15	179.8 (5)
C5—C4—C3	118.6 (3)	C19—C17—C18	119.8 (3)
C5—C4—H4A	120.7	C19—C17—S3	123.6 (3)
C3—C4—H4A	120.7	C18—C17—S3	116.6 (3)
N1—C5—C4	121.0 (3)	N7—C18—C17	179.1 (4)
N1—C5—H5A	119.5	C17—C19—C20	119.3 (3)
C4—C5—H5A	119.5	C17—C19—S4	123.1 (3)
N2—C6—C3	177.4 (5)	C20—C19—S4	117.6 (3)
N1—C7—H7A	109.0	N8—C20—C19	179.9 (4)
N1—C7—H7B	109.7	S3—Cu1—S2	178.59 (4)
H7A—C7—H7B	109.5	S3—Cu1—S1	87.63 (4)
N3—C8—C9	121.0 (3)	S2—Cu1—S1	90.96 (4)
N3—C8—H8A	119.5	S3—Cu1—S4	90.93 (4)
C9—C8—H8A	119.5	S2—Cu1—S4	90.48 (4)
C10—C9—C8	119.0 (3)	S1—Cu1—S4	178.56 (4)
C10—C9—H9A	120.5	C1—N1—C5	120.9 (4)
C8—C9—H9A	120.5	C1—N1—C7	119.6 (4)
C9—C10—C9^i^	119.1 (4)	C5—N1—C7	119.5 (4)
C9—C10—C11	120.4 (2)	C8^i^—N3—C8	120.9 (4)
C9^i^—C10—C11	120.4 (2)	C8^i^—N3—C12	119.5 (2)
N4—C11—C10	178.2 (5)	C8—N3—C12	119.5 (2)
N3—C12—H12A	109.5	C13—S1—Cu1	101.21 (13)
N3—C12—H12B	109.5	C15—S2—Cu1	101.68 (13)
H12A—C12—H12B	109.5	C17—S3—Cu1	101.52 (13)
N3—C12—H12C	109.5	C19—S4—Cu1	100.90 (13)
H12A—C12—H12C	109.5		

Symmetry codes: (i) *x*, −*y*+1/2, *z*.

### Hydrogen-bond geometry (Å, °)

**Table:**

*D*—H···*A*	*D*—H	H···*A*	*D*···*A*	*D*—H···*A*
C1—H1A···N8^ii^	0.93	2.60	3.533 (6)	179
C2—H2A···N7^ii^	0.93	2.44	3.309 (6)	156
C5—H5A···N4^iii^	0.93	2.36	3.247 (6)	159
C8—H8A···N2^iv^	0.93	2.48	3.196 (5)	134
C9—H9A···N5^v^	0.93	2.51	3.297 (4)	143

Symmetry codes: (ii) −*x*+1, *y*+1/2, −*z*+1; (iii) −*x*+1, *y*+1/2, −*z*; (iv) *x*−1, *y*−1, *z*; (v) −*x*+1, *y*−1/2, −*z*.
